# Structural insights into human caspase-9 CARD assembly modes driven by conserved arginine hotspots

**DOI:** 10.1016/j.yjsbx.2026.100163

**Published:** 2026-09-14

**Authors:** Swasti Rawal, Christoph Grininger, Sem Peijnenborgh, Patrick David Fischer, Tobias Madl, Tea Pavkov-Keller

**Affiliations:** a Research Unit Integrative Structural Biology, Medicinal Chemistry, Otto Loewi Research Center, Medical University of Graz, 8010 Graz, Austria; b Institute of Molecular Biosciences, University of Graz, 8010 Graz, Austria; c BioTechMed-Graz, 8010 Graz, Austria

**Keywords:** Caspase-9, CARD, Cell-death, cancer, Apoptosis, Crystal structure

## Abstract

Caspase-9 (C9) is a key initiator of the intrinsic apoptotic pathway and contains an N-terminal caspase activation and recruitment domain (CARD) that mediates homotypic and heterotypic interactions. While previous studies have shown that C9^CARD^ can form filamentous assemblies under specific conditions, its intrinsic structural properties and interaction modes below saturation concentration remain incompletely understood. Here, we present a 2.4 Å resolution crystal structure of the isolated human C9^CARD^, revealing a sulfate-mediated dimer-of-dimers assembly involving a conserved arginine-rich surface. Notably, these residues overlap with those previously shown in filament formation and in interactions with APAF-1^CARD^, indicating reuse of a common interaction hotspot across distinct assembly states. Complementary biophysical analyses show that C9^CARD^ remains predominantly monomeric under physiological conditions, while sulfate induces changes in its hydrodynamic properties. Compared to WT C9^CARD^, a triple-arginine mutant (R10E/R52E/R56E) shows different sulfate-dependent behavior and abolishes formation of higher-order assemblies. Additionally, WT C9^CARD^ exhibits reversible temperature-dependent transitions between filamentous and droplet-like assemblies. Finally, AlphaFold3 predictions suggest that filament-forming propensity and electrostatically regulated higher-order assemblies may extend to other human CARDs. Together, our findings suggest a model in which a conserved electrostatic interaction motif enables multiple structurally distinct assembly modes of C9^CARD^. This work highlights conserved electrostatic interaction modes in CARD-CARD association and suggests that environmental conditions can modulate their assembly behavior.

## Highlights

• The crystal structure of isolated human Caspase-9 CARD (C9^CARD^) reveals a sulfate-mediated dimer-of-dimers arrangements.
• The observed interfaces reuse a conserved arginine-rich hotspots also present in C9^CARD^ filaments and in C9^CARD^/APAF-1^CARD^ complex.
• The triple-arginine (R10E/R52E/R56E) mutant diminishes the sulfate-dependent hydrodynamic response and impairs higher-order C9^CARD^ assembly.
• C9^CARD^_undergoes a reversible, temperature-dependent phase transition between liquid-like droplets and helical filamentous assemblies._
• AlphaFold3 Predictions suggest that filament-forming potential may extend to other human CARD-containing proteins.

## Graphical abstract

**Figure:**
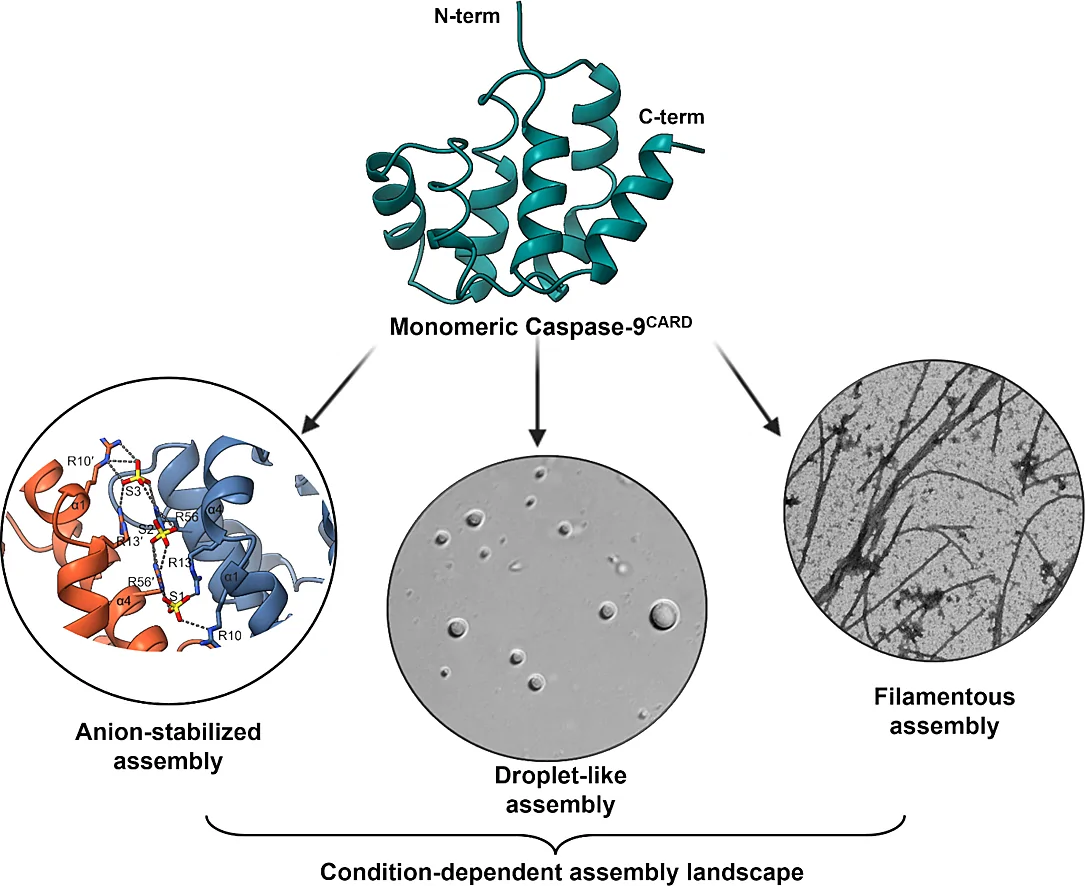

## 1 Introduction

Caspase-9 (C9), a cysteine-aspartic protease, plays a key role as an initiator of the intrinsic apoptotic pathway and regulates physiological and pathological cell death processes by activating downstream effector caspases, such as caspase-3 (C3) and caspase-7 (C7) (Julien and Wells, 2017; Shi, 2002). At the molecular level, C9 is a 416-residue protein that is sequentially comprised of a caspase activation and recruitment domain (CARD), a 40-residue linker, and a protease domain (Riedl and Salvesen, 2007; Riedl and Shi, 2004).

The N-terminal CARD domain belongs to the death-domain fold (DDF) superfamily, which is characterized by a six-helix bundle fold. DDF superfamily is composed of four subfamilies, named as Death Domain (DD), Pyrin domain (PYD), Caspase activation and recruitment domain (CARD), and the Death Effector Domain (DED) (Wu et al., 2025; Zhou et al., 2021; Park et al., 2007). The structures of DDFs are highly conserved among the different family members (Zhou et al., 2021). DDF-containing proteins are the main components in apoptotic and inflammatory pathways, with any dysregulation due to alternative splicing or genetic mutations being strongly associated with immunodeficiency and autoimmune dysfunctions. For instance, mutations in the Fas gene that disrupt death-inducing signaling complex (DISC) formation are associated with autoimmune lymphoproliferative syndrome (ALPS) (Park et al., 2007; Huoh and Hur, 2022; Rieux-Laucat et al., 1995; Hoffman et al., 2001). Moreover, DDF-containing proteins often use multiple interfaces to interact with themselves and other DDFs, leading to complex modes of homo- and hetero-oligomerization or aggregation. One mode of DDF oligomerization leads to the formation of filaments, which have been detected both *in vitro* and *in vivo*. Importantly, filament formation was shown to be essential for the activation of certain caspases, including Caspase-1 (C1) and Caspase-8 (C8) (Lu et al., 2016; Fox et al., 2021; Li et al., 2018). However, other CARD-containing caspases have not been reported to form filaments, and instead form soluble oligomers during their activation. The structural plasticity of DDFs, ranging from insoluble filaments, soluble oligomers, and monomers, is an interesting feature as there is considerable structural similarity between different DDF family members, as shown for the C1 and C9 CARDs. Notably, the purified C1^CARD^ forms filaments (Lu et al., 2016) whereas the C9^CARD^ is reported to be a soluble monomer at basal concentration (Shiozaki et al., 2002; Hu et al., 2014; Qin et al., 1999; Li et al., 2017).

Under basal conditions, C9 is present as an inactive zymogen and requires activation by the apoptosome, a heptameric complex comprising APAF-1 and cytochrome C (Qin et al., 1999; Rodriguez and Lazebnik, 1999; Li et al., 1997). Once activated, C9 cleaves and activates downstream effector caspases, triggering the onset of cell death through caspase-mediated proteolysis of cellular proteins. Failure to activate C9 enables the evasion of programmed cell death, as occurs in various forms of cancer, uncontrolled inflammatory responses, and neurodegenerative disorders (Brown and Attardi, 2005; Hengartner, 2000; Johnstone et al., 2002; Mattson, 2000). Previous structural studies till date have mostly focused on C9^CARD^ in a hetero-oligomeric complex with APAF-1 or as part of the apoptosome, providing molecular insight into heterotypic interactions (Hu et al., 2014; Qin et al., 1999; Li et al., 2017; Cheng et al., 2016; Su et al., 2017). Recently, it has been demonstrated that C9^CARD^ oligomerizes and forms filamentous assemblies *in vitro* under physiological conditions at a saturation concentration (*C*_sat_) or the maximum concentration at which the protein remains soluble at given solution conditions, driven primarily by the electrostatic interactions (Rawal et al., 2025). However, the structural features and self-association behavior of the isolated C9^CARD^ below its saturation concentration (*C*_sat_) remain poorly understood. Another way of oligomerization of many DDF-containing proteins is the formation of visible puncta or foci within cells, a property of the biomolecular condensates generated through liquid-liquid phase separation (LLPS) (Huoh and Hur, 2022). Recent studies have shown that phase separation of NLR family pyrin domain containing 6 (NLRP6) with viral RNA, both *in vitro* and in cells, can initiate the formation of the NLRP6:ASC (apoptosis-associated speck-like protein containing a CARD) inflammasome (Shen et al., 2021).

To further elucidate the structural features and possible self-association modes of C9^CARD^ below its *C*_sat_, we determined the crystal structure of the isolated domain in the absence of its binding partner. We further investigated whether the sulfate-mediated assembly observed in a crystal structure is detectably populated in solution using complementary DLS and NMR measurements and assessed the contribution of the arginine-rich surface using a triple-arginine (R10E/R52E/R56E) mutant. We also show that C9^CARD^ undergoes a reversible, temperature-dependent phase transition between higher-order structured filaments and liquid-like droplets, as observed by differential interference contrast (DIC) microscopy. Finally, we extended our analysis by comparing filament-forming propensities across other human CARDs to place the observed C9^CARD^ behavior within the broader context of CARD-containing protein family. Overall, our study focuses on the structural and physicochemical basis of different C9^CARD^ assembly states however, the physiological relevance of the observed assemblies remain unclear and is not investigated in the present study.

## 2 Results

CARD-containing proteins often use multiple interfaces to interact with themselves or with some other CARDs, leading to complex modes of homo- and hetero- oligomerization (Park et al., 2007; Huoh and Hur, 2022; Qin et al., 1999; Ferrao and Wu, 2012). However, at low concentration (100 μM), under physiological conditions, human C9^CARD^ predominantly behaves as a folded, soluble monomer , as shown by SEC (Fig. 1A, Supplementary Figs. 1A-B), CD spectroscopy (Supplementary Fig. 10) and a well-resolved NMR spectrum (Fig. 1B). Previous study reported that the C9^CARD^ oligomerizes by itself and forms higher-order structured filaments *in vitro* at pH 7.0 (Rawal et al., 2025). The propensity to form filaments was enhanced under low-salt or acidic conditions, suggesting a significant role for electrostatic interactions in driving C9^CARD^ self-association.

**Fig. 1:**
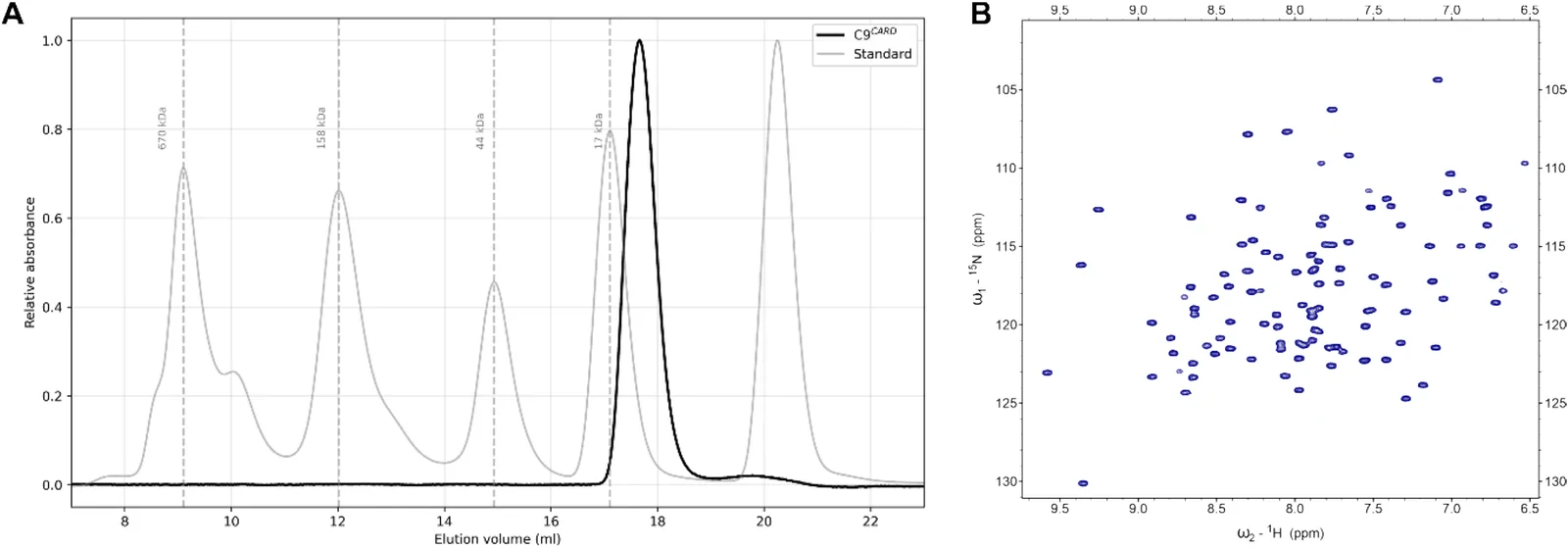
Characterization of WT human C9^CARD^ in solution: (A) Overlay of the size-exclusion chromatography (SEC) elution profile of C9^CARD^ (black) with a molecular weight standard (gray) recorded on a Superdex 200 Increase 10/300 column equilibrated in 50 mM HEPES, 200 mM NaCl, pH 7.5. Dashed lines indicate the elution positions of the standard proteins (Thyroglobulin, Gamma-globulin, Ovalbumin, Myoglobin) with their corresponding molecular weights (670 kDa, 158 kDa, 44 kDa, 17 kDa) and elution volume (9.1 mL, 12.02 mL, 14.93 mL, 17.1 mL). C9^CARD^ elutes as a single symmetric peak at 17.65 mL. (B) 2D ^1^H—^15^N HSQC spectrum 100 μM of ^15^N-labeled C9^CARD^ in 25 mM MES, 50 mM NaCl, 5 mM TCEP, 0.5 mM EDTA, pH 6.5 buffer at 25 °C, showing well-dispersed resonances consistent with a folded and soluble protein.

All currently available C9^CARD^ structures have been determined in presence of its binding partner APAF-1^CARD^ (PDBs: 3YGS, 3JUY, 4RHW, 5WVC, 5WVE) (Hu et al., 2014; Qin et al., 1999; Li et al., 2017; Cheng et al., 2016; Su et al., 2017). To gain structural insight into the intrinsic assembly properties of C9^CARD^, we determined its crystal structure without binding partner at 2.4 Å resolution. Clusters of needle-like crystals were obtained at 15 mg/ml C9^CARD^ concentration with cross-seeding in SG1 H-37 condition containing 1.5 M Lithium sulfate, 0.1 M Sodium HEPES (pH 7.5). The detailed X-ray data collection and refinement parameters are summarized in Supplementary Table 1. The asymmetric unit contains four C9^CARD^ monomers arranged in a dimer-of-dimers configuration with interfaces stabilized by negatively charged sulfate ions and positive arginine residues (Fig. 2A). The four chains show a compact quaternary arrangement with a root mean square deviation (RMSD) of 0.46 Å, with each monomer maintaining the canonical six-helical bundle fold characteristic of the CARDs or DDF-containing protein (Supplementary Fig. 2). Although 1.5 M lithium sulfate represents a non-physiological crystallization condition, the observed sulfate-mediated contacts highlight a structurally accessible interaction mode involving a conserved arginine-rich surface (R10, R13, R15, R52, R56). Mutational analysis of several of these residues overlap with positions previously shown to influence C9^CARD^ filament formation, supporting the relevance of this region as a recurrent and structurally accessible interaction surface.

**Fig. 2:**
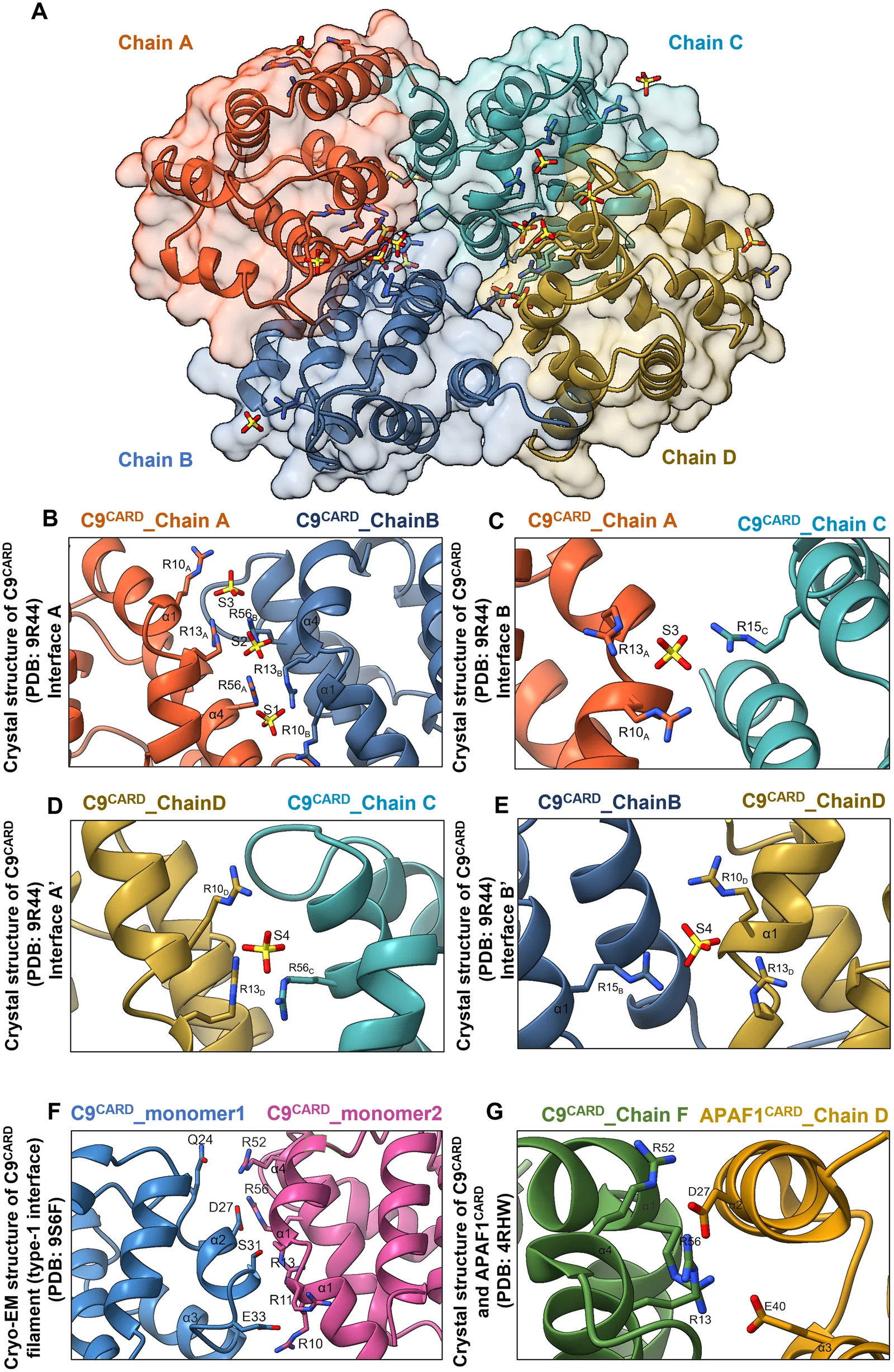
Conserved arginine-mediated interfaces in C9^CARD^ oligomerization: (A) Asymmetric unit of the crystal structure containing four C9^CARD^ monomers, arranged as a dimer of dimers with interfaces stabilized by sulfate ions (the four chains are shown in different colors). Interacting residues at the interface with negatively charged sulfate ions are shown with sticks. (B-E) Zoomed in view of the (B) interface A (between chain A and B), (C) interface B (between chain A and C), (D) interface A′ (between chain C and D), and (E) interface B′ (between chain B and D) respectively, showing key interacting residues stabilizing the CARD-CARD interaction in presence of sulfate ions (S1, S2, S3, S4). (F) Type-I interface of the C9^CARD^ filament (PDB: 9S6F) shows the same key arginine residues (R10, R13, R15, R52, R56) form canonical salt bridges with acidic residues on neighboring subunits, stabilizing the helical assembly. This interaction mode matches the contact points observed in the anion-mediated crystal structure interface. (G) Interface of the previously solved C9^CARD^-APAF-1^CARD^ complex (PDB: 4RHW). Again, R13, R52, and R56 of C9^CARD^ interacts with the negatively charged residues of adjacent APAF-1^CARD^, forming the characteristic Type-I death-domain specific interface.

Two distinct interface types are present in the crystal structure: interfaces A and A′ involve comparable sets of residues, as do interfaces B and B′. Interfaces A and A′ are symmetry-related and comprises chains A-B and C—D, respectively, whereas interfaces B and B′ include chains A-C and B—D. All four interfaces are mediated by electrostatic interactions between an arginine-rich surface patch and sulfate ions, but they differ in the number and spatial arrangement of sulfate molecules. Whereas interface A contains three sulfate ions, interface A′ features only a single, central sulfate ion that is coordinated by the same set of arginine residues. The interface A is stabilized by charge-mediated interaction between helix α1 and α4 arginines (R10_A_, R13 _A_, R15_A_, R56_A_) of chain A and helix α1 and α4 arginines (R10_B_, R13_B_, R52_B_, R56_B_) of chain B with sulfate ions S1, S2 and S3 (Fig. 2B, Supplementary Fig. 3 A–C). Interface A′ is maintained by interaction between helix α1 arginines (R10_D_ and R13_D_) of chain D and arginine of helix α4 (R56_C_) of chain C with the sulfate ion S4 (Fig. 2D, Supplementary Fig. 3D). Interface B is similarly stabilized by electrostatic interactions via the charged side chains of arginine and the negative sulfate ions. This interface is formed by the interaction between helix α1 and α4 arginines (R10_A_ and R13_A_) of chain A and arginine of helix α1 (R15_C_) of chain C with the sulfate ion S3 (Fig. 2C, Supplementary Fig. 3C). Interface B′ is stabilized by interaction between helix α1 arginines (R10_D_ and R13_D_) of chain D and arginine of helix α1 (R15_B_) of chain B with the sulfate ion S4 (Fig. 2E, Supplementary Fig. 3D). The buried surface area (BSA) for interface A, A′, B and B′ correspond to 489.2 Å^2^, 494.2 Å^2^, 486.3 Å^2^ and 558.0 Å^2^ respectively (Supplementary Table 2).

Together, these interfaces define a sulfate-mediated dimer-of-dimers arrangement centred on a conserved arginine-rich surface. The overlap of these residues with interaction sites observed in previously determined C9^CARD^ structures identify this region as a recurrent and structurally accessible interaction surface across distinct structural contexts.

Furthermore, the crystal structure of the isolated human C9^CARD^ shows a conserved fold with the C9^CARD^ within the filamentous assembly with an RMSD value of 0.68 Å (Supplementary Fig. 4 A–C) and with previously determined structures of C9^CARD^ in presence of APAF-1^CARD^ with an RMSD values in the range of 0.54 Å to 0.58 Å (Supplementary Fig. 5 A–D). This structural conservation indicates that C9^CARD^ retains a highly similar fold across different assembly states, suggesting that self-association occurs without major conformational rearrangements of the monomer. Interestingly, the residues involved in making the crystal contact of C9^CARD^ (R10, R13, R15, R52, and R56) (PDB: 9R44) overlap with the type-I interface residues described for the C9^CARD^ filament (R10, R13, R52, and R56) (PDB: 9S6F) (Fig. 2F). Moreover, the interacting interfaces for the C9^CARD^ and APAF-1^CARD^ complex (PDB: 4RHW) involves a subset of same arginine residues of C9^CARD^ (R13 and R52) (Fig. 2G). These observations further support the involvement of a conserved arginine-rich surface in mediating C9^CARD^ self-association across different structural contexts.

Direct superposition of isolated C9^CARD^ (PDB 9R44), filament-associated C9^CARD^ (PDB 9S6F), and APAF-1-bound C9^CARD^ (PDB 4RHW) further demonstrates conservation of the overall CARD fold. However, the arginine side chains show local differences in orientation and spatial positioning (Supplementary Fig. 6). This suggests that the observed structural differences arise primarily from rearrangement of solvent-exposed arginine side chains rather than from major conformational changes of the C9^CARD^ fold.

### 2.1 Sulfate modulates the solution properties of C9^CARD^

To examine whether the sulfate-mediated assembly observed in the crystal structure is detectably populated in solution, we first investigated sulfate-dependent changes in C9^CARD^ using dynamic light scattering (DLS). WT C9^CARD^ showed a progressive shift toward larger apparent hydrodynamic radii at elevated sulfate concentrations (Supplementary Fig. 7A). Because DLS does not resolve molecular stoichiometry and is particularly sensitive to larger scattering species, these measurements indicate sulfate-dependent changes in particle size but do not establish the formation of a discrete oligomeric state. Furthermore, no additional peak appeared in the SEC chromatogram when using 250 μM of C9^CARD^ in sulfate containing buffers.

To further assess how sulfate alters the solution-state environment of C9^CARD^, ^1^H—^15^N HSQC spectra were recorded in the absence and presence of 100 mM sulfate. Comparison of the spectra revealed widespread sulfate-dependent changes in peak positions and relative signal intensities across the HSQC spectral profile (Supplementary Fig. 8), indicating that the local chemical environment of C9^CARD^ is altered under sulfate-containing conditions. In addition, one-dimensional ^1^H NMR spectra also showed a pronounced sulfate-dependent reduction in overall protein signal intensity (Supplementary Fig. 9A). This attenuation was not restricted to a subset of peaks but was observed across the entire protein resonance envelope. The increased line broadening is consistent with a reduction in the soluble C9^CARD^ population due to its incorporation into a larger heterogenous assembly exhibiting slower tumbling. Given this evidence for a potentially NMR-invisible population, we next asked whether the population that remains NMR-visible also shows altered rotational behavior. TRACT (TROSY for Rotational Correlation Times) measurements were therefore performed in the absence and presence of 500 mM sulfate. The estimated rotational correlation time increased from 5.2 ± 0.8 ns to 7.0 ± 0.7 ns upon sulfate addition (Supplementary Fig. 9C—D), corresponding to an increase of about 35%. This modest increase indicates slower rotational diffusion of the observable population and is consistent with an increase in effective hydrodynamic size. Importantly, however, the magnitude of the change does not support wholesale conversion of the NMR-visible population into a substantially larger, well-defined oligomer. To probe whether the sulfate-dependent loss of NMR signal reflected exchange with a sparsely populated NMR-invisible state on the millisecond timescale, ^15^N chemical exchange saturation transfer (CEST) measurements were performed. No additional exchange state was detected under the conditions tested (exemplary peak shown in Supplementary Fig. 9B), arguing against the presence of an exchanging, NMR-invisible state. Taken together, the DLS and NMR data indicate that sulfate shifts C9^CARD^ toward larger and more heterogeneous solution states. DLS revealed an increase in apparent particle size, while NMR showed residue-specific chemical shift perturbations, substantial signal reduction consistent with partial loss into poorly observable species, and a modest increase in the rotational correlation time of the remaining NMR-visible population. However, ^15^N CEST did not detect a substantially populated exchanging state on the millisecond timescale probed. Thus, although sulfate promotes changes consistent with increased association of C9^CARD^, the data does not support accumulation of a stable higher-order assembly state corresponding to the crystallographic dimer-of-dimers.

To directly assess the contribution of the arginine-rich region to C9^CARD^ assembly behavior, we analyzed a triple-arginine mutant (R10E/R52E/R56E), designed based on the crystallographic interface. In DLS measurements, the mutant displayed a markedly smaller sulfate-dependent shift in apparent hydrodynamic radius compared with WT C9^CARD^ (Supplementary Fig. 7B), indicating an attenuated response to elevated sulfate concentrations. Far-UV CD spectra of C9^CARD^ WT and triple-arginine mutant showed comparable profiles characteristic of a predominantly α-helical protein, indicating that the mutations did not cause a major disruption of the overall secondary structure (Supplementary Fig. 10). Furthermore, negative-stain EM showed extensive ordered filamentous assemblies for WT C9^CARD^, whereas the mutant predominantly remained soluble and displayed amorphous aggregate (Supplementary Fig. 11). Together, these observations support a contribution of the arginine-rich region to the ability of C9^CARD^ to access higher-order assembly states.

### 2.2 Temperature-dependent transitions between filamentous and droplet-like C9^CARD^ assemblies

Based on the condition-dependent C9^CARD^ self-association behavior, such as pH, salt and protein concentration (Rawal et al., 2025) as well as on presence of anions, we next investigated how temperature affects its oligomerization behavior. At 25 °C, C9^CARD^ is soluble below its *C*_sat_. In contrast, after two weeks of storage at 4 °C we observed the assembly of C9^CARD^ (∼600 μM concentration) into long, straight filament bundles. These filament bundles were consistent with the previously reported filamentous assemblies observed by electron microscopy (Rawal et al., 2025). The sample tube exhibited a hydrogel-like appearance suggesting the formation of an extended filament network (Fig. 3C). Upon incubation of the same sample at room temperature (RT) for few minutes, all C9^CARD^ filament bundles dissolved and reorganized into droplet-like assemblies (Fig. 3D) as visualized by differential interference contrast (DIC) microscopy. The corresponding sample became clear and transparent consistent with reduced filament formation and enhanced solubility at higher temperature. Interestingly, re-incubation of the same sample at 4 °C for few minutes, rapidly restored filament assembly (Fig. 3E) with the reappearance of bundled structures and a hydrogel-like appearance, indicating a reversible transition between filamentous and droplet-like states.

**Fig. 3:**
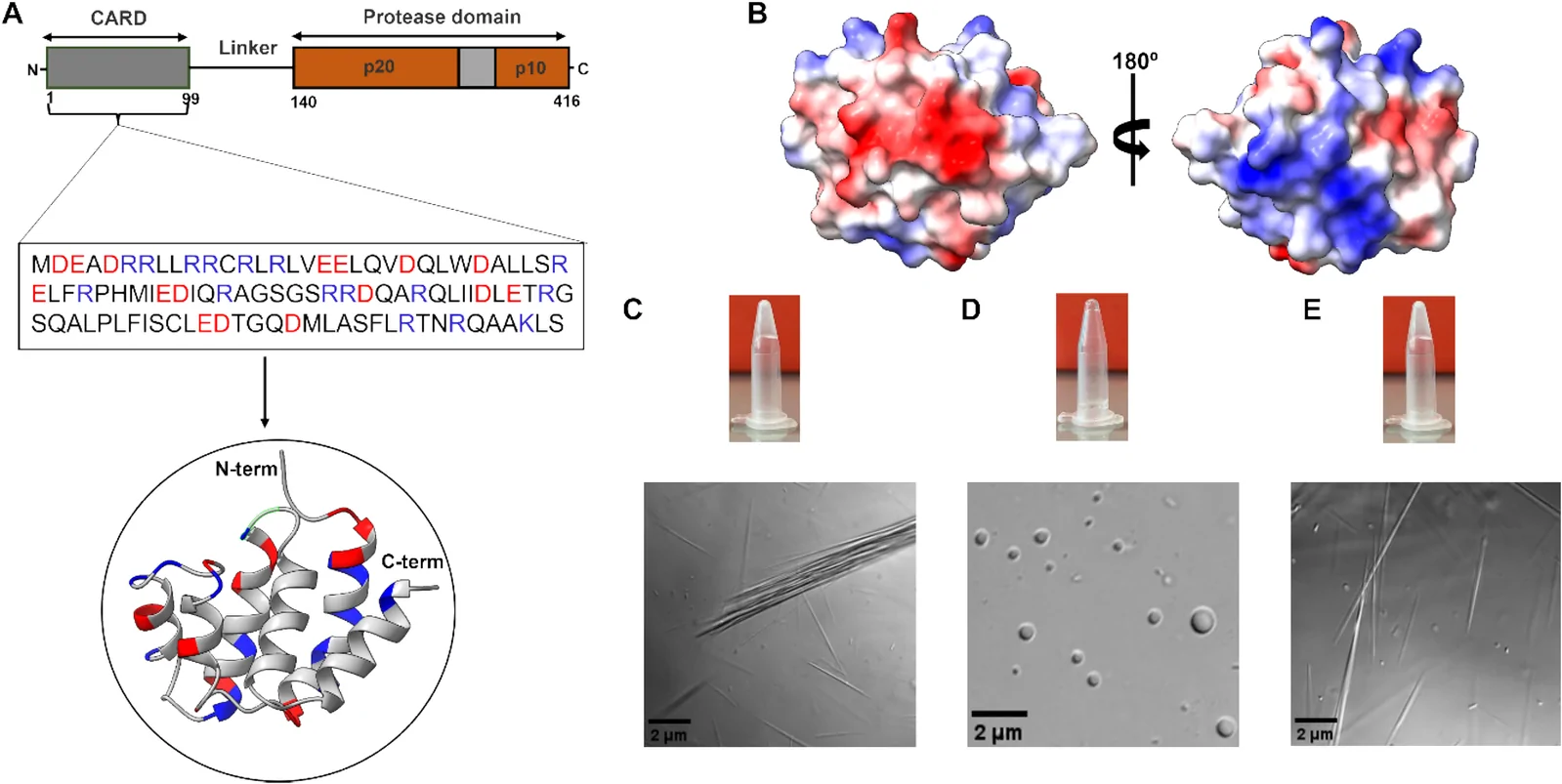
Temperature-dependent reversible phase transition of C9^CARD^: (A) Structural arrangement of full-length human C9, depicting the N-terminal caspase activation and recruitment domain (CARD), followed by a short flexible linker, and the C-terminal protease domain comprising of a large (p20) and a small (p10) subunit. The N-terminal CARD domain mediates interaction with APAF-1^CARD^, enabling recruitment to the heptameric apoptosome, activating the protease domain that cleaves the downstream effector caspases to initiate further signaling. Charged residues are highlighted and color coded onto the structure (positively charged residues-blue; negatively charged residues-red) (B) Electrostatic potential of C9^CARD^ highlighting charged patches on the surface. (C) Differential interference contrast (DIC) micrograph of C9^CARD^ (∼600 μM) at 4 °C, showing structured filamentous assemblies. The corresponding sample tube above displays hydrogel-like morphology, highlighting filament network formation. (D) Representative DIC micrograph of the previous sample at room temperature, C9^CARD^ forms droplet-like assemblies. The corresponding sample tube shows a clear soluble protein, showing disappearance of filaments. (E) DIC micrograph of the same re-incubated sample at 4 °C, showing restored filament assembly, and the hydrogel-like sample appearance in the corresponding tube image. (For interpretation of the references to color in this figure legend, the reader is referred to the web version of this article.)

In contrast to WT C9^CARD^, triple-arginine mutant showed neither apparent filament formation at 4 °C nor droplet-like assemblies following incubation at room temperature under the conditions described above (Supplementary Fig. 12). Together with the DLS and negative-stain EM results, this observation further supports a contribution of the arginine-rich region to higher-order C9^CARD^ assembly.

### 2.3 Filament-forming propensity of CARD-containing proteins based on AlphaFold3 (AF3) predictions

CARD-CARD interaction is of vital importance for the modulation of signaling cascade such as inflammasome formation during an inflammatory response or apoptosome assembly during an intrinsic lethal signal. To explore whether structural features observed in C9^CARD^ may extend to other CARD domains, we used AlphaFold3 (AF3) (Abramson et al., 2024) to assess potential self-association tendencies. Using AF3 webserver, we ran predictions for other CARDs which are not yet known to form filaments. We used all the filament-forming CARDs with the experimental information available for benchmarking. Fig. 4A shows the heatmap based on the AF3-derived interface predicted template modeling (ipTm) values. For each CARD, the best model from three independent AF3 runs was selected and the mean ipTm value was plotted as a function of oligomeric state (number of subunits). The top 9 rows depict the CARDs with known filament forming behavior and experimentally determined structural information. Below that are the CARDs which are not yet reported to be filamentous. Based on the predictions, CARD11^CARD^, CARD16^CARD^, CARD17^CARD^, CARD18^CARD^, C2^CARD^, CRADD^CARD^, RAIDD2^CARD^ shows an ipTm score of >0.5 and the per residue aligned error (PAE) plot showed confidence with 12 subunits, indicating potential of assembling into higher-order assemblies (Fig. 4C—I). Consistent with these observations, negative-stain EM showed that APAF-1^CARD^ formed predominantly assembled into amorphous aggregates rather than structured filaments (Supplementary Fig. 11A). However, C9^CARD^ readily assembled into extensive filament network confirming its self-oligomerization ability (Supplementary Fig. 11B). These observations suggest that AF3-based predictions may provide a useful starting point to gather information about the protein oligomerization propensity, followed by experimental validation of the *in-silico* predictions.

**Fig. 4:**
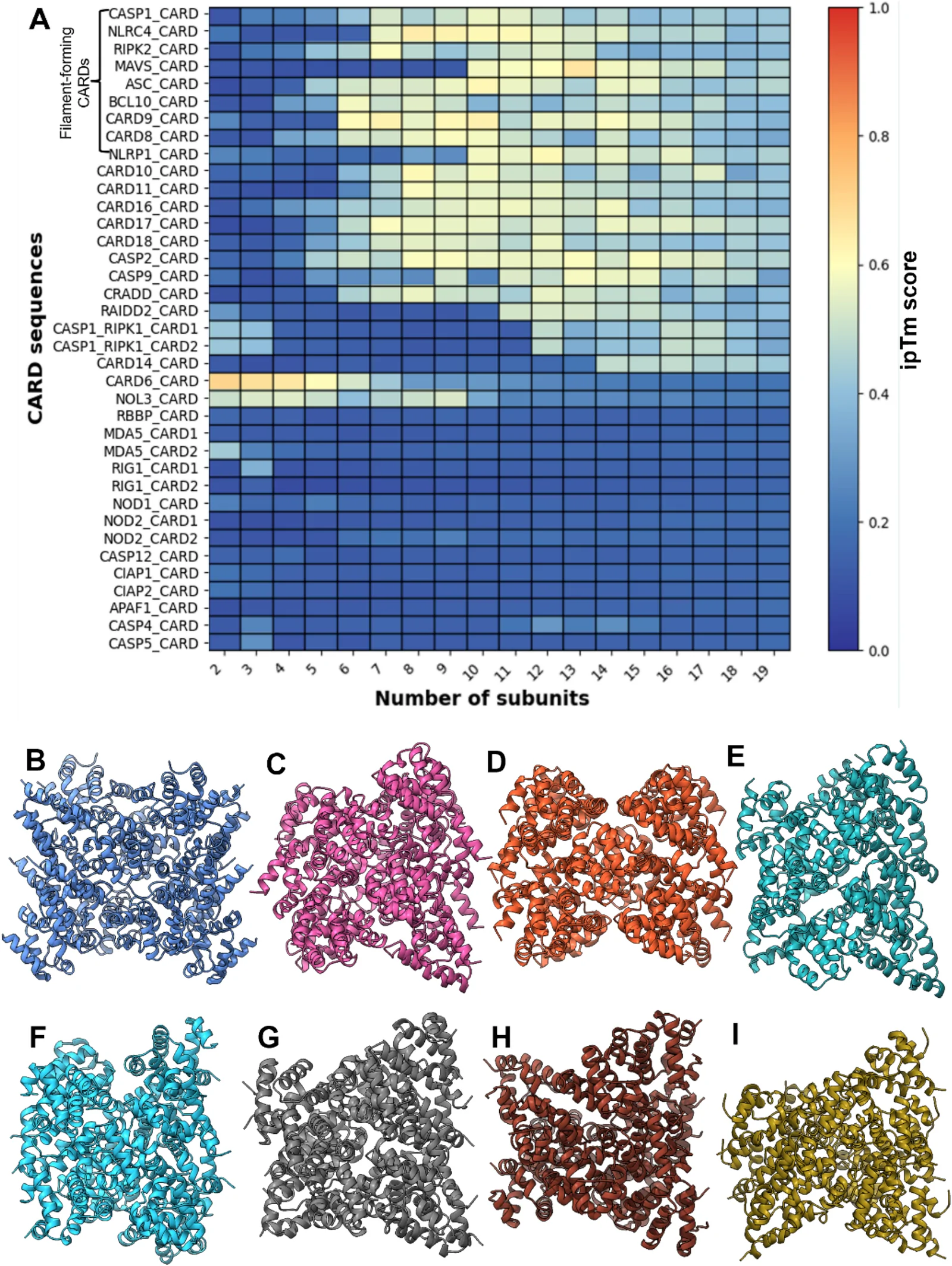
AlphaFold3 (AF3) prediction of human CARDs: (A) AlphaFold3 prediction of human CARD oligomerization, plotting the number of predicted subunits on x-axis against an average interface predicted template modeling (ipTm) scores of all three seeds used for prediction. (B) Experimental structure of WT C9^CARD^ filaments (PDB: 9S6F) at 3.3 Å resolution, illustrating a two-fold symmetry (Rawal et al., 2025). AF3 predicted filament assemblies of human CARDs with 12 subunits (C) CRADD^CARD^ (D) Caspase-2^CARD^ (E) RAIDD2^CARD^ (F) CARD16^CARD^ (G) CARD18^CARD^ (H) CARD17^CARD^ (I) CARD11^CARD^.

## 3 Discussion

CARD domains are key protein-protein interaction modules that mediate the assembly of signaling complexes through homo- and heterotypic interactions (Wu et al., 2025; Park et al., 2007). Despite their conserved six-helical fold, CARD-containing proteins exhibit diverse oligomerization behaviors, ranging from monomers and soluble oligomers to higher-order filamentous assemblies (Wu et al., 2025; Huoh and Hur, 2022; Lu et al., 2016; Fox et al., 2021; Li et al., 2018). Understanding how this structurally conserved fold supports such diversity in assembly remains an important question in the field.

The role of C9 as an initiator caspase depends not only on its catalytic activity but also on the higher-order organization of its CARD domain. Through CARD-CARD interactions, C9 is recruited to the apoptosome via APAF-1^CARD^ and activated in an induced-proximity manner (Park et al., 2007; Chao et al., 2005; Salvesen and Dixit, 1999). Despite its relatively small size and conserved six-alpha helical fold, the CARD domain plays a central role in regulating C9 activation (Li et al., 2017; Rodriguez and Lazebnik, 1999). Previous studies have primarily focused on C9^CARD^ in complex with APAF-1^CARD^ within the apoptosome (Hu et al., 2014; Qin et al., 1999; Li et al., 2017; Cheng et al., 2016; Su et al., 2017). Recent work demonstrated thar C9^CARD^ can self-assemble into structuted filaments under specific solution conditions (Rawal et al., 2025), raising the possibility that this domain may access multiple assembly states beyond its canonical apoptosome-associated conformation. Therefore, we used C9^CARD^ as a model system to perform structural analysis of its condition-dependent assembly behavior.

In this work, we investigated the structural and physicochemical basis of condition-dependent C9^CARD^ assembly states below its *C*_sat_, focusing on how this domain adopts distinct interaction states under different conditions. (Fig. 5). Under basal conditions, C9^CARD^ is predominantly monomeric, whereas crystallization in high sulfate condition captured a sulfate-mediated dimer-of-dimers centered on a conserved arginine-rich surface. Previous research has shown that C9^CARD^ assembles into structured filaments at *C*_sat_ or under low-salt or acidic conditions (Rawal et al., 2025). Structural comparison with filament-associated and APAF-1-bound C9^CARD^ demonstrates that the overall six-helix fold and spatial organization of this positively charged surface are highly conserved across distinct interaction contexts. (Hu et al., 2014; Qin et al., 1999; Li et al., 2017; Cheng et al., 2016; Su et al., 2017). Reuse of this surface provides a structural basis by which C9^CARD^ may participate in distinct homo- and heterotypic interactions without major conformational rearrangement of the monomer. Whether other negatively charged cellular components can engage the same electrostatic surface remains an interesting possibility for future investigation.

**Fig. 5:**
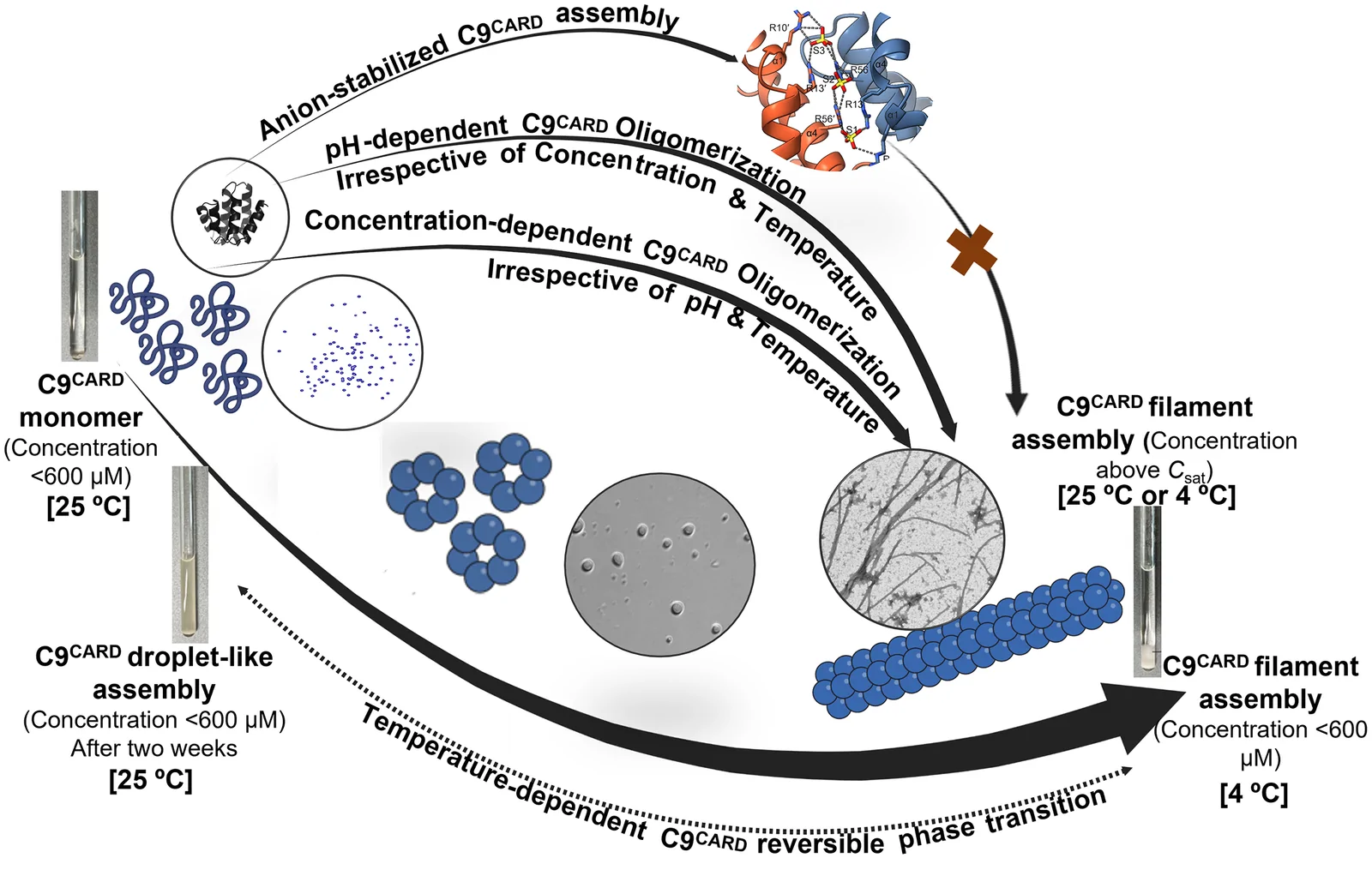
Schematic working model of the C9^CARD^ assembly landscape: (Bottom) Schematic representation of the temperature-dependent reversible phase transition behavior of C9^CARD^ between structured filaments and droplet-like assemblies. At concentrations ∼600 μM, under physiological conditions, and 25 °C, C9^CARD^ predominantly behaves as a monomer. Upon prolonged incubation at lower temperature (4 °C), these droplets transformed into higher-ordered structured filaments. The process is reversible, highlighting a temperature-dependent C9^CARD^ phase transition from soluble monomers to droplet-like and ultimately to filamentous assembly. Insets illustrate representative DIC microscopy images of droplets and electron microscopy images of filament bundles. (Top) Schematic representation of condition-dependent C9^CARD^ filament formation. Increasing protein concentration or changes in solution conditions (anion presence and pH), promote self-association and filament formation. Above *C*_sat_, C9^CARD^ shows a strong tendency to form structured higher-order filamentous assemblies irrespective of pH and temperature conditions. (parts of the figure were prepared in BioRender. Rawal, S. (2026) https://Biorender.com/dh88aqt).

Importantly, the sulfate-mediated assembly observed in the crystal structure likely represents a structurally accessible interaction mode rather than a dominant physiological state. The solution measurements further define the limits of this structural interpretation. Sulfate produced clear changes in the DLS profiles and NMR chemical shifts, demonstrating that C9^CARD^ is responsive to changes in its ionic environment. The modest increase in rotational correlation time measured by TRACT and the absence of an additional substantially populated state in the CEST analysis further claims the absence of a stable sulfate-induced higher-order oligomer under the conditions tested. Thus, the crystallographic dimer-of-dimers is best interpreted as a sulfate-stabilized, structurally accessible assembly state rather than a dominant solution species.

It is important to note that the crystallographic dimer-of-dimers was captured under a combination of conditions that strongly favor intermolecular encounters, including high protein concentration, high sulfate concentration, prolonged equilibration, and the crystallization environment. The observed assembly may therefore require the combined effect of several physicochemical parameters for stabilization and need not represent a substantially populated species in dilute solution. Nevertheless, the sulfate-dependent DLS and NMR changes demonstrate that C9^CARD^ responds to sulfate in solution. Additionally, the mutational analysis supports functional relevance of the broader arginine-rich region for C9^CARD^ assembly behavior. The triple-arginine mutant retained a predominantly α-helical secondary structure but showed a strongly reduced sulfate-dependent DLS response and abolished formation of higher-order assemblies in negative-stain EM and DIC microscopy. These observations indicate that the positively charged surface contributes to C9^CARD^ assembly behavior.

Furthermore, WT C9^CARD^ displayed a reversible temperature-dependent transition between filamentous bundles at 4 °C and droplet-like assemblies at room temperature. Although 4 °C is not physiologically relevant, the reversibility of this behavior illustrates the sensitivity of C9^CARD^ higher-order assembly to environmental conditions. A key parameter governing this phase transition is the initial protein concentration. In a previous study, C9^CARD^ was saturated until it reached *C*_sat_ driving the system directly into an aggregated, filamentous state (Rawal et al., 2025). However, when we use C9^CARD^ concentration below its *C*_sat_, we do not force immediate filament formation. Instead, C9^CARD^ can dynamically exchange between filamentous and droplet-like assembly. The absence of comparable filamentous or droplet-like assemblies for the triple-arginine mutant under the tested conditions further suggests that the arginine-rich region contributes to this condition-dependent higher-order assembly behavior. Such behavior suggests that, under appropriate cellular conditions (*e.g.* locally increased concentration, changes in ionic strength, or association with lipids, ATP or RNA), C9^CARD^ may undergo analogous droplet-like-to-filament transitions, similar to anion-associated phase behaviors described for other CARD-containing proteins (Shen et al., 2021; Huang et al., 2022; Cao et al., 2025; Chai et al., 2025; Pizzuto et al., 2022; Elliott et al., 2018). Together with the anion-mediated interfaces described above, these observations emphasize that relatively small physicochemical changes can shift C9^CARD^ between distinct assembly states.

C9^CARD^ has an overall near-neutral net charge, with an isoelectric point of approximately pH 6.5. Although it is not a canonical low-complexity disordered domain, its amino-acid sequence is compositionally biased: nearly 50% of the residues are Ala, Arg, Asp, or Leu (Fig. 3A). The primary sequence is considerably enriched in charged residues (33% of all positions), with an equal distribution of positively charged (15 Arg, 1 Lys) and negatively charged residues (9 Asp, 7 Glu) (Fig. 3A–B). Together, these features suggest that the conserved charged surface of C9^CARD^ is consistent with a strong sensitivity to changes in the electrostatic environment. Thus, both sequence composition and structurally recurrent charged surfaces position C9^CARD^ as an electrostatically responsive interaction module.

Several CARD- and DDF-containing proteins have been reported to form cellular foci, condensates or filamentous assemblies, including NLRP6^CARD^, MAVS^CARD^, RIP2^CARD^, ASC^CARD^ and caspase-2^CARD^ (Li et al., 2018; Shen et al., 2021; Tenthorey et al., 2017; Diebolder et al., 2015; Yang et al., 2018; Xu et al., 2014; Gong et al., 2018; Dick et al., 2016; Sborgi et al., 2015; Su et al., 2008; Ge et al., 2024). These observations illustrate the broader capacity of death-domain-fold (DDF) proteins to access diverse supramolecular states. Whether the droplet-like C9^CARD^ assemblies observed here correspond to a physiologically relevant cellular state, and whether such assemblies participate in filament nucleation or signaling, remain open questions requiring direct cellular and biophysical investigation.

Given that several CARD-containing proteins exhibit similar foci and higher-order assemblies, we next asked whether filament-forming propensity might be a more general property of CARD domains. To explore this, we employed the AF3 webserver to predict multimeric CARD-CARD assemblies across a range of human CARDs. The predictions revealed that a subset of CARDs exhibit a helical filament-forming propensity, suggesting that oligomerization and higher-order assembly could be a general feature of the CARD family. Experimental structure determination and functional validation will be required to elucidate whether, and under which conditions, these predicted CARD-CARD assemblies correspond to physiologically significant CARD-mediated supramolecular signaling complexes.

Taken together, our data position C9^CARD^ as a versatile oligomerization module whose electrostatically driven assemblies are highly responsive to environmental conditions. In a broader context, we propose a working model in which C9^CARD^ samples a range of assembly states depending on physicochemical conditions (Fig. 5). Sulfate stabilizes one such arrangement in the crystal and perturbs C9^CARD^ in solution without generating a detectable substantially populated discrete oligomer. At higher protein concentrations and under temperature-dependent conditions, C9^CARD^ can additionally access filamentous and droplet-like assemblies. Mutational analysis further suggests that the arginine-rich region is a key player in this varied electrostatic interaction modes. Instead of representing discrete functional endpoints, these states may reflect a continuous assembly landscape governed by electrostatic interactions and local environmental cues. Linking specific structural interfaces to distinct supramolecular states might provide a framework for understanding how CARD-mediated interactions can be dynamically regulated. Future structural and cellular studies on other CARDs, guided by AF3-based predictions, will be important to determine how general these assembly principles are and how they relate to CARD-dependent signaling pathways.

While this study provides structural and biophysical insight into the assembly behavior of C9^CARD^, several limitations should be considered. The assembly states described here are characterized under *in vitro* conditions, and their physiological relevance remains to be established. The sulfate-mediated interfaces observed in the crystal structure likely represent an accessible interaction mode highlighting a conserved arginine-rich surface, rather than a dominant solution state, as discrete oligomers were not resolved under the tested conditions. Although increasing anion concentration promotes self-association, the underlying species remain difficult to define using the approaches applied here. Finally, while reversible transitions between filamentous and droplet-like states illustrate the different interaction modes of C9^CARD^, the molecular nature and functional relevance of these intermediate states require further investigation. Finally, the AF3-predicted assemblies require direct experimental validation. Nonetheless, the combined structural, NMR, biophysical and mutational data identify a conserved arginine-rich surface as an important contributor to the condition-dependent assembly behavior of C9^CARD^.

## 4 Materials and methods

### 4.1 C9^CARD^ expression and purification

The gene encoding the C9^CARD^ domain subcloned into the pET-29b(+) vector (kanamycin resistance) to generate an N-terminal His₆-SUMO fusion construct was generously provided by Dr. T. Reid Alderson. The expression and purification were performed as described in Rawal et al., 2025 (Rawal et al., 2025). Briefly, the protein was expressed in E. coli BL21(DE3) cells and purified by Ni^2+^-affinity chromatography (50 mM Tris-HCl, 500 mM NaCl, 20 mM imidazole, 2 mM DTT, pH 7.5) followed by His₆-SUMO tag removal by treatment with Ulp1R3 protease at 25 °C for 15 min at room temperature and size-exclusion chromatography on a Superdex 75 Increase 10/300 column (GE Healthcare) equilibrated with 20 mM HEPES, 200 mM NaCl, 0.5 mM EDTA, and 1 mM TCEP (pH 7.5). The protein was concentrated using a 3 kDa molecular-weight cutoff concentrator Amicon® Ultra. Protein purity was confirmed by SDS-PAGE, and concentration was determined spectrophotometrically at 280 nm using a molar extinction coefficient calculated with the ExPASy ProtParam tool (Gasteiger et al., 2005). The final yield of purified C9^CARD^ was approximately 30 mg per litre of culture.

Triple-arginine mutant was subcloned into His_6_-protein A pETZ2_1a vector (GenScript), containing a tobacco etch virus (TEV) protease cleavage site downstream of the protein A tag. Protein expression and purification were performed as described above except the His_6_ protein A fusion tag was cleaved by overnight TEV protease treatment at 4 °C.

### 4.2 Analytical size exclusion chromatography (SEC)

Purified C9^CARD^ was analyzed by analytical size-exclusion chromatography using a Superdex 200 Increase 10/300 GL column equilibrated in 50 mM HEPES, 200 mM NaCl, pH 7.5. Protein was loaded at a concentration of 1 mg/ml, and elution was monitored by absorbance at 280 nm. The same measurements were repeated in the buffer containing sulfate (100–500 mM). To estimate the apparent molecular weight of C9^CARD^, the column was calibrated under identical buffer conditions using a Bio-Rad gel filtration standard. A calibration curve was generated by plotting the logarithm of the molecular weight of the standard proteins against their corresponding elution volumes, and the apparent molecular weight of C9^CARD^ was determined from the resulting linear regression. The linear regression was performed with scipy.stats from the SciPy python package and plotted with Matplotlib.

### 4.3 NMR spectroscopy

To produce ^15^N-labeled C9^CARD^ sample, M9 minimal medium was used with 1 g/L of ^15^NH_4_Cl (Sigma) and 6 g/L of *d*-glucose (Roth). The purification was performed as outlined above. NMR sample was prepared in 95% H_2_O/5% D_2_O in a total sample volume of 500 μL. Two-dimensional (2D) ^1^H—^15^N heteronuclear single-quantum coherence (HSQC) NMR spectra was recorded at 298 K on a 600-MHz Bruker Avance Neo NMR spectrometer equipped with a TXI 600S3 probe head. Measurements were performed using 250 μM ^15^N-labeled C9^CARD^ in 25 mM MES, 50 mM NaCl, 5 mM TCEP, pH 6.5 in the absence and presence of 100 mM sulfate to assess sulfate-dependent changes at residue-level resolution. The NMR spectrum was processed using Bruker *TopSpin* version 4.4.0 (Bruker Biospin, Ettlingen, Germany).

One-dimensional ^1^H NMR spectra were recorded using 250 μM C9^CARD^ in 25 mM MES, 50 mM NaCl, 5 mM TCEP, pH 6.5, in the absence and presence of 500 mM sulfate to assess sulfate-dependent changes in the overall spectral profile and signal intensity. Spectra were acquired at 298 K and processed identically to allow direct comparison of relative signal intensities between conditions.

To evaluate sulfate-dependent changes in the rotational behavior of the NMR-visible C9^CARD^ population, TRACT (TROSY for Rotational Correlation Times) (Favier and Brutscher, 2019; Rennella and Brutscher, 2013) measurements were performed under sulfate-free and 500 mM sulfate conditions using 250 μM ^15^N-labeled C9^CARD^ in 25 mM MES, 50 mM NaCl, 5 mM TCEP, pH 6.5 buffer. Relaxation data were fitted to obtain the transverse relaxation rates (R_2_^+^) and (R_2_^−^), and the difference between these rates, ΔR_2_, was used to estimate the rotational correlation time (τ_c_). ^15^N chemical exchange saturation transfer (CEST) (Vallurupalli et al., 2012) measurements were performed in the presence of 500 mM sulfate using 700 μM ^15^N-labeled C9^CARD^ in 25 mM MES, 50 mM NaCl, 5 mM TCEP, pH 7.5 buffer, to probe for potentially low-populated conformational or assembly states. CEST profiles were generated by plotting the normalized peak intensity (I/I_0_) as a function of the ^15^N saturation frequency. Saturation was applied for 400 ms using a B1 field of 25 Hz over a range of 103-134 ppm in 0.5 ppm increments.

### 4.4 Dynamic light scattering (DLS)

DLS measurements were performed using an XtalDLS instrument (XtalConcepts) in a microbatch crystallization plate. Purified C9^CARD^ (WT) and triple-arginine-mutant were measured at a final concentration of 250 μM in a total volume of 1 μl per condition. Measurements were performed at room temperature without sulfate and in the presence of sodium sulfate concentrations ranging from 100 to 800 mM. For each condition, 80 scans were recorded at 2-min intervals. Intensity-weighted particle size distributions were obtained and plotted as intensity *versus* hydrodynamic radius using GraphPad Prism version 5.01.

### 4.5 Circular dichroism (CD) spectroscopy

Far-UV circular dichroism (CD) spectra of WT C9^CARD^ and the triple-arginine mutant were measured with 10 μM of protein in 25 mM phosphate buffer, pH 7.5 using a 0.1 cm pathlength cuvette (Chirascan Plus spectrometer Applied Photophysics). CD spectra were recorded at room temperature over the wavelength range 185-260 nm. Five individual scans were recorded and averaged, and the corresponding buffer spectrum was subtracted for baseline correction. The spectra were smoothed using a Savitzky-Golay filter and the resulting ellipticity values were converted to mean residue ellipticity (MRE) using the respective protein molecular weights, concentrations, path length, and number of residues. The processed spectra were plotted using Matplotlib.

### 4.6 Negative-staining transmission electron microscopy (ns-TEM)

A 400-mesh carbon-coated copper grid (Science Services, Germany) was glow-discharged using a PELCO easiGlow™ unit (Ted Pella Inc., USA). 10 μL of purified sample (buffer: 25 mM MES, 200 mM NaCl, 0.5 mM EDTA, 2 mM TCEP, pH 7.5 or 5.5) was applied to the glow-discharged grid and allowed to adsorb for 1 min. Excess sample was blotted off with filter paper, followed by addition of 10 μL of 1% (*w/v*) uranyl acetate staining solution. After 1 min of adsorbtion, excess stain was carefully removed by blotting and the grid was left to air-dry for few minutes. Samples were imaged using a Tecnai G2 FEI transmission electron microscope (Thermo Fisher Scientific Inc., USA) operated at an acceleration voltage of 120 keV and equipped with an Ultrascan 1000 CCD camera (Gatan AMETEK, Germany).

### 4.7 X-ray crystallography

The monomeric C9^CARD^ (WT) peak fractions were collected from the final SEC run in 20 mM HEPES, 200 mM NaCl, 0.5 mM EDTA, 1 mM TCEP, pH 7.5 buffer. The purified protein was dialyzed against the crystallization buffer 20 mM Hepes, 50 mM NaCl, 2 mM TCEP, and pH 7.5 buffer and was concentrated to 15 mg/mL using a 3 kDa Amicon Ultra-15 Centricon (Millipore Merck, Darmstadt, Germany). Initial crystallization conditions were setup using commercially available standard screens: JCSG + and ShotGun1 (SG1) (Molecular Dimensions, Calibre Scientific, Rotherham, UK). The protein concentration used were 7 mg/mL and 15 mg/mL. Crystallization trials were carried out using an ORYX 8 robot (Douglas Instruments, Hungerford, UK) with the sitting-drop vapor diffusion approach in 96-well plates (Swissci AG, Neuheim, Switzerland). The drops contained 0.5 μL of C9^CARD^ and 0.5 μL of reservoir solution and were equilibrated against 35 μL of reservoir solution. Small tiny crystals were observed in SG1 H-9 condition. These crystals were used to prepare a seed stock using a small magnetic bead. An additional 0.1 μL of a 1:10 and 1:100 of 15 mg/ml diluted seeding stock was applied for seeding experiments. Crystallization plates were incubated at 16 °C. Crystallization condition containing 1.5 M Lithium sulfate, 0.1 M Sodium HEPES, and pH 7.5 in SG1 Screen, (Molecular Dimensions, Calibre Scientific, Rotherham, UK). Needle-like crystals appeared within 4 weeks and were frozen in liquid nitrogen without cryo protection for the diffraction experiments. C9^CARD^ (WT) crystallization information is summarized in Supplementary Table 3. For the diffraction experiment, a single crystal was fetched using a cryoloop and flash froze in liquid nitrogen (LN_2_). For the triple-arginine mutant, same crystallization steps were performed as for WT using SG1 crystal screen. No crystals were obtained.

Initial crystal screening was performed with an in-house dual port system equipped with a MetalJet X-ray source (Excillium, Kista, Sweden), a D8 Venture X-ray diffractometer (Bruker, Billerica, USA) and a Photon III detector (Bruker, Billerica, USA). A final dataset with a resolution of 2.4 Å, space group *P*2_1_ 2_1_ 2_1_ with cell constants of 39.92, 86.39, 129.96 Å and 90°, 90° and 90° was collected at the ID23–2 beamline of the ESRF in Grenoble (France) at 100 K using a DECTRIS EIGER X 9 M detector. Data processing was done using XDS (Kabsch, 2010) and data scaling was done using Aimless (Evans and Murshudov, 2013). The structure was solved by molecular replacement using human C9^CARD^ (PDB-ID 3ygs; Chain P) as template in Phenix (Adams et al., 2010). The structure was built using Coot and refined using phenix.refine. Waters were placed by enabling the ‘update waters’ option in Phenix. Data collection and processing statistics are summarized in Supplementary Table 1.

The final analysis and validation of the refined model was done using PDBePISA, MolProbity (Davis et al., 2007), and PDB validation report. Data collection and refinement statistics were obtained using the Phenix Table 1. All structure rendering and structure alignments were generated using ChimeraX (UCSF ChimeraX version: 1.7.1) (Goddard et al., 2018). Interfaces were studied with the PDBepisa server. RMSD values were calculated using the ChimeraX ‘matchmaker’ structural analysis tool. The atomic coordinates and structure factors have been deposited in the RCSB Protein Data Bank (Rose et al., 2012) under the accession code PDB ID 9R44.

### 4.8 Temperature-dependent DIC microscopy

For the phase separation, recombinantly purified C9^CARD^ WT at a concentration of 650 μM in 50 mM Tris, 50 mM NaCl, 2 mM TCEP, 20 mM imidazole, 0.04% (*w/v*) NaN3, pH 7.5 was incubated at 4 °C for two weeks without any agitation. After the incubation period, sample turns into a thick hydrogel state which consist of protein filaments. A 22 × 40 mm rectangular glass coverslip (Biotrend Chemikalien GmbH, Cologne, Germany) was taken to put 20 μL of hydrogel sample, which was then mounted onto an inverted Zeiss Observer Z1 (Zeiss, Jena, Germany) fluorescence microscope equipped with a Plan Apochromat 63× oil immersion M27 objective with differential interference contrast (DIC) nosepiece. The sample was visualized for 5 min at room temperature (RT) with an interval of 5 s using a motorized, 12 V 100 W Halogen lamp. The formation of liquid droplets was induced by over time with incubation at RT. To assess the temperature dependence of C9^CARD^ (WT) phase behavior, the same coverslip sample was transferred to 4 °C and monitored over time. All micrographs were processed using ImageJ 1.53a software (National Institutes of Health, Bethesda, US), where brightness and contrast was adjusted by applying a linear contrast image enhancement. The triple-arginine mutant was examined using the same imaging workflow at 4 °C and room temperature.

### 4.9 AlphaFold3 (AF3) calculations

We curated the amino acid sequence of all CARD-containing human protein in fasta format. To accurately predict the interactions of all curated protein in multichain protein complexes (multimers) and generate structural models, we used AF3 webserver (Abramson et al., 2024). For the AF3 modeling of CARD filaments, we first need to identify how many subunits are “optimal” for the calculation. Since this information was not known beforehand, we had to determine the number empirically. To get the quantitative information about the optimal subunit number, we started predictions by varying the number of subunits from 1 to 20 for each CARD and observed how interface predicted template modeling score (ipTm) changes as a function of the number of CARD protomers. In AF3, the ipTM score quantifies the predicted accuracy of the interfaces between chains, on a scale from 0 (low confidence) to 1 (high confidence) for the relative arrangement and contacts of interacting subunits. For each CARD, five models (model IDs 0–4) were generated. AF3 uses a random seed value in its sampling procedure. To probe the robustness of the predictions, we therefore performed the calculations with three different random seeds (10, 99, and 250). We used CARDs with available experimental evidence of filament formation as benchmarks. For the quantitative heatmap, we took, for each CARD and oligomeric state, the model with the highest ipTM score across the three seeds and then calculated the mean of ipTM values. This average ipTM was plotted as a function of the predicted CARD oligomeric state. The visualization was done using Matplolib library from Python. The resulting predicted models were rendered using the ChimeraX (UCSF ChimeraX version: 1.7.1) (Goddard et al., 2018).

## CRediT authorship contribution statement

**Swasti Rawal:** Writing – review & editing, Writing – original draft, Visualization, Validation, Project administration, Methodology, Investigation, Formal analysis, Data curation, Conceptualization. **Christoph Grininger:** Writing – review & editing, Visualization, Resources, Methodology, Investigation. **Sem Peijnenborgh:** Formal analysis, Visualization, Writing – review & editing. **Patrick David Fischer:** Formal analysis, Investigation, Methodology, Validation, Visualization, Writing – review & editing. **Tobias Madl:** Writing – review & editing, Supervision, Resources, Project administration, Funding acquisition, Formal analysis, Conceptualization. **Tea Pavkov-Keller:** Writing – review & editing, Validation, Supervision, Resources, Project administration, Methodology, Funding acquisition, Formal analysis, Conceptualization.

## Declaration of competing interest

The authors declare that they have no known competing financial interests or personal relationships that could have appeared to influence the work reported in this paper.

## Data Availability

X-ray data collected at the ESRF are available at DOI: 10.15151/ESRF-ES-1299724102. The crystal structure and corresponding structure factors are deposited to the PDB: 9R44 (DOI: 10.2210/pdb9r4x/pdb).
