# Supplementary material for: Structural insights into human caspase-9 CARD assembly modes driven by conserved arginine hotspots
Source: J Struct Biol X. 2026 Sep 14;14:100163. doi: 10.1016/j.yjsbx.2026.100163 (PMC13626777; doi:10.1016/j.yjsbx.2026.100163)
Supplement: Supplementary file 1 [file mmc1.docx]

Structural insights into human caspase‑9 CARD assembly modes driven by conserved arginine hotspots

Swasti Rawal^a,*^, Christoph Grininger^b^, Sem Peijnenborgh^a^, Patrick David Fischer**^a^**, **Tobias Madl^a,c^, Tea Pavkov-Keller^b,c,*^**

^a^ Research Unit Integrative Structural Biology, Medicinal Chemistry, Otto Loewi Research Center, Medical University of Graz, 8010 Graz, Austria

^b^ Institute of Molecular Biosciences, University of Graz, 8010 Graz, Austria

^c^ BioTechMed-Graz, 8010 Graz, Austria

* corresponding authors: tea.pavkov@uni-graz.at; [rawalswasti@gmail.com](mailto:rawalswasti@gmail.com)

**Supplementary Figure 1. Size-exclusion chromatography analysis of C9^CARD^: (A)** Chromatogram of C9^CARD^ at 100 µM concentration in 25 mM HEPES buffer, 200 mM NaCl, pH 7.5, monitored at 280 nm. The elution profile indicates predominantly monomeric behavior. Inset shows the SDS-PAGE profile of C9^CARD^ showing a band at ~12 kDa. **(B) Calibration curve generated from the elution volumes of the standard proteins plotting log_10_(molecular weight) against elution volume. The calculated molecular weight (MW) from the linear regression for C9^CARD^ is ~ 12 kDa. The black triangle depicts the measured elution volume against the** log_10_(MW) of the theoretical MW of the C9^CARD^.

**Supplementary Figure 2.** Structural overlay of the four C9^CARD^ monomers of the asymmetric unit of the final refined crystal structure (deposited under PDB accession ID: 9R44), yielding a root-mean-square deviation (RMSD) of 0.46 Å, indicating high level of structural conservation across all chains.

**Supplementary Figure 3. Crystal structure of WT human C9^CARD^: (A-D)** Contact maps showing the interaction details between residues of individual chain with the sulfate ions are generated by using LigPlot+ (version v2.3.1).

**Supplementary Figure 4. Structural comparison of experimental WT C9^CARD^ from crystal and Cryo-EM filament reconstructions: (A)** Chain D of C9^CARD^ crystal structure. **(B)** Cryo-EM derived WT C9^CARD^ filament (green) (deposited under PDB accession ID: 9S6F). Both the structures show canonical six-helix bundle fold. **(C)** Structural overlay of the WT C9^CARD^ crystal structure (gold) with the WT C9^CARD^ filament monomer (green), showing high degree of structural fold conservation with an RMSD value of 0.68 Å.

**Supplementary Figure 5. Structural comparison of all experimental C9^CARD^ structures:** Structural overlay of human C9^CARD^ from the PDB: 9R44 (gold) with PDB: 5JUY (cyan) **(A)**; PDB: 4RHW (green) **(B)**; PDB: 5WVE (pink) **(C)**; and PDB: 3YGS (purple) **(D).** The RMSD values are 0.58 Å, 0.58 Å, 0.54 Å, and 0.55 Å respectively, depicting a highly conserved structural fold irrespective of the presence or absence of the binding partner.

**Supplementary Figure 6. Structural comparison of C9^CARD^ in distinct assembly states:** Superposition of C9^CARD^ from the isolated crystal structure (PDB 9R44; Blue), the C9^CARD^-APAF-1^CARD^ complex (PDB 4RHW; Green), and the C9^CARD^ filament (PDB 9S6F; Pink). The conserved arginine residues R10, R13, R15, R52, and R56 are shown as sticks to highlight their relative positions and side-chain orientations across the three structures.

**Supplementary Figure 7. Sulfate-dependent changes in the hydrodynamic radius of C9^CARD^: (A) Dynamic light scattering (DLS) size distributions of C9^CARD^ WT recorded in the absence of sulfate or in the presence of 400 mM to 800 mM sulfate, showing a progressive shift toward larger apparent hydrodynamic radii above 500 mM sulfate concentration. (B) Triple-arginine m**utant displays a strongly reduced sulfate-dependent increase in apparent hydrodynamic radius across the tested conditions.

**Supplementary Figure 8. Sulfate-induced changes in C9^CARD^:** Overlay of ^1^H-^15^N HSQC spectra of 200 µM C9^CARD^ in 25 mM MES buffer, pH 6.5, in the absence of sulfate (blue) and in the presence of 100 mM sulfate (orange).

**Supplementary Figure 9. NMR analysis of C9^CARD^ in the presence of sulfate:** **(A)** Overlay of 1D ^1^H NMR spectra of C9^CARD^ recorded in the absence (red) and presence of 500 mM sulfate (green). **(B)** Representative ^15^N CEST profile of C9^CARD^ in the presence of 500 mM sulfate, showing the normalized intensity ratio (I/I_0_) as a function of ^15^N saturation frequency for the indicated peak. **(C-D)** TRACT relaxation profiles of C9^CARD^ recorded in the absence of sulfate (C) and in the presence of 500 mM sulfate (D). The fitted (R_2_^+^) and (R_2_^-^) relaxation rates were used to calculate (ΔR_2_) and estimate the rotational correlation time (τ_c_). The corresponding τ_c_ values were 5.2 ± 0.8 ns without sulfate and 7.0 ± 0.7 ns in the presence of 500 mM sulfate.

**Supplementary Figure 10.** **Far-UV circular dichroism (CD) spectra of C9^CARD^ WT and triple-arginine mutant:** Far-UV CD spectra of WT C9^CARD^ (solid line) and the triple-arginine mutant (dashed line) display comparable spectral profiles characteristic of a predominantly α-helical secondary structure. MRE- mean residue ellipticity.

**Supplementary Figure 11. Negative-stain electron microscopy of C9^CARD^ & APAF1^CARD^: (A)** Negative staining electron micrograph of WT C9^CARD^, showing higher order filamentous assemblies. **(B)** Negative staining electron micrograph of triple-arginine mutant, assembling into amorphous aggregates instead of structured filaments. **(C)** Negative staining electron micrograph of APAF1^CARD^, forming amorphous aggregates instead of ordered structured assemblies.

**Supplementary Figure 12. Temperature-dependent DIC microscopy of the triple-arginine mutant:** Representative DIC micrographs of the triple-arginine mutant at **(A)** 4 °C and **(B)** the same sample incubated at room temperature. No apparent droplet-like or higher-order filament assemblies were observed.

**Supplementary Table 1.** X-ray data collection and structure refinement. Statistics for the highest-resolution shell are shown in parentheses.

| **Contents** | **C9^CARD^ (WT) values** |
| --- | --- |
| **PDB code** | **9R44** |
| **Data collection & processing** |  |
| Data collection facility | Synchrotron ESRF Beamline ID23-2 |
| Detector | DECTRIS EIGER X 9M |
| Wavelength | 0.87313 |
| Resolution range (Å) (low to high) | 38.16 - 2.46  (2.55 - 2.46) |
| Space group | *P* 2_1_ 2_1_ 2_1_ |
| No. of molecules in the asymmetric unit | 4 |
| Unit cell (Å; º) | a=39.92, b=86.39, c=129.96; α=β=γ=90˚  α=90, β=90, γ=90 |
| Total reflections | 111739 (11138) |
| Unique reflections | 17006 (1660) |
| Multiplicity | 6.6 (6.7) |
| Completeness (%) | 99.9 (99.9) |
| Mean I/sigma(I) | 9.4(2.3) |
| Wilson B-factor | 34.67 |
| CC1/2 | 0.995 (0.770) |
| R_merge_ | 0.173 (0.930) |
| R_pim_ | 0.073 (0.386) |
| **Refinement Statistics** |  |
| Reflections used in refinement | 16998 (2072) |
| Reflections used for R-free | 1398 (170) |
| R_work_ | 0.2063 (0.2551) |
| R_free_ | 0.2647 (0.2943) |
| Number of non-hydrogen atoms | 3430 |
| Macromolecules | 3214 |
| Ligands | 92 |
| Solvent | 124 |
| Protein residues | 396 |
| **Model Geometry** |  |
| RMSD bonds (Å) | 0.002 |
| RMSD angles (º) | 0.46 |
| **Ramachandran distribution** |  |
| Ramachandran favored (%) | 98.97 |
| Ramachandran allowed (%) | 1.03 |
| Ramachandran outliers (%) | 0.00 |
| Rotamer outliers (%) | 0.00 |
| Clashscore | 3.05 |
| **Average B-factor (A^2^)** | 44.12 |
| Macromolecules | 43.80 |
| Ligands | 58.78 |
| Solvent | 41.47 |

**Supplementary Table 2.** PDBePISA analysis of crystallographic interfaces in the C9^CARD^ anion-stabilized assembly.

| **Interface** | **Chair pair** | **Buried surface area (Å²)** | **Δ^i^G**  **(kcal/mol)** | **Δ^i^G**  **P-value** | **Hydrogen bonds** | **Salt bridges** |
| --- | --- | --- | --- | --- | --- | --- |
| Interface A | A-B | 489.2 | 0.5 | 0.478 | 3 | 0 |
| Interface B | A-C | 486.3 | -2.1 | 0.230 | 6 | 2 |
| Interface A′ | C-D | 494.2 | -1.4 | 0.274 | 4 | 0 |
| Interface B′ | B-D | 558.0 | -4.4 | 0.128 | 10 | 0 |

**Supplementary Table 3.** Crystallization of human C9^CARD^

| Method | *Vapour diffusion, sitting drop* |
| --- | --- |
| Plate type | 96-well plates |
| Data collection temperature (K) | 100 |
| Protein concentration (mg/ml) | 15 |
| Buffer composition of protein solution | 20 mM Hepes, 50 mM NaCl, 2 mM TCEP, and pH 7.5 |
| Buffer composition of crystal screen | 1.5 M Lithium sulfate, 0.1 M Sodium HEPES, and pH 7.5 |
| Volume of reservoir (µl) | 35 |
